# Does the use of supraglottic device in rabbits cause less injury than other airway management devices?

**DOI:** 10.18849/ve.v7i4.608

**Published:** 2022-12-02

**Authors:** Jasmine Gheini+, Sanaa Zaki+

**Keywords:** AIRWAY MANAGEMENT, ANAESTHESIA, ENDOTRACHEAL TUBE, FACE MASK, INJURY, INTUBATION, LARYNGEAL MASK, RABBIT, SUPRAGLOTTIC AIRWAY DEVICE, TRAUMA, V-GEL®

## Abstract

PICO question

In rabbits, undergoing general anaesthesia, does the placement of a v-gel® device result in less airway trauma compared to the use of other airway management devices?

Clinical bottom line

Category of research question

Treatment

The number and type of study designs reviewed

Three papers were critically appraised, two blinded randomised experimental trial studies and one randomised crossover experimental trial study

Strength of evidence

Weak

Outcomes reported

One blinded randomised trial study demonstrated that the trauma to the upper airways of rabbits during anaesthesia is not significantly different between the use of v-gel® and an endotracheal tube. The other blinded randomised trial study demonstrated that the trauma to the upper airway caused by endotracheal intubation is significantly more than that caused by v-gel® placement. The randomised crossover trial study demonstrated that v-gel® placement causes more significant compression to the larynx compared to a laryngeal mask or face mask

Conclusion

The current evidence suggests that use of the v-gel® in rabbits causes less trauma / injury to the airways compared to placement of an endotracheal tube but not compared to the use of a laryngeal or face mask. However, based on the low number and quality of published studies, this evidence is weak, and better-quality studies are required to support the routine use of v-gel® over other airway devices in rabbits. While v-gel® may be a safer alternative for securing airways in rabbits compared to endotracheal intubation, knowing the injuries this device can cause to the upper airways is useful for managing rabbits during post anaesthesia recovery.

This Knowledge Summary has reviewed the available evidence on the use of a SGAD (v-gel®) in rabbit anaesthesia. Since writing a new design of a single use supraglottic airway device (SGAD) has been introduced; currently there is no published evidence on whether this new device has an impact on the risk of injury

How to apply this evidence in practice

The application of evidence into practice should take into account multiple factors, not limited to: individual clinical expertise, patient’s circumstances and owners’ values, country, location or clinic where you work, the individual case in front of you, the availability of therapies and resources.

Knowledge Summaries are a resource to help reinforce or inform decision making. They do not override the responsibility or judgement of the practitioner to do what is best for the animal in their care.

## Clinical scenario

As a veterinarian and owner of a small animal clinic with a high rabbit caseload, you would like to improve the protocols used for rabbit anaesthesia in your practice. You have recently heard of a new supraglottic airway device specific for rabbits named v-gel®, and you are considering introducing v-gel® in your clinic. You are wondering if this device is safer and causes less trauma in rabbits undergoing general anaesthesia compared to your current practice of using face masks for short procedures and endotracheal tubes for longer procedures.

## The evidence

This critical appraisal identified three relevant studies. Two blinded randomised experimental trials (Comolli et al., 2020; and Engbers et al., 2017) and one randomised crossover experimental trial (Wenger et al., 2017).

Comolli et al. (2020) used a blinded randomised experimental trial approach to study on healthy female New Zealand White rabbits and determine if the placement of v-gel® would lead to faster, easier secure airways with less histological trauma compared to endoscopic endotracheal intubation with cuffed endotracheal tubes. The second blinded randomised experimental trial, Engbers et al. (2017) studied on healthy adult New Zealand White rabbits to determine similar goals to Comolli et al. (2020) however, the endotracheal intubation was preformed with a blind technique. Wegner et al. (2017) preformed a randomised crossover experimental trial study on healthy female New Zealand White rabbits to compare v-gel®, endotracheal tube, face mask and laryngeal mask in regard to ease of application, time of application and quality of seal.

Although randomised trials are considered to represent a moderate level of evidence, due to the small number of studies relevant to this PICO, and the study’s limitations such as small sample size, the findings from these three trials provided only a weak level of evidence that v-gel® devices cause less airway trauma when compared to other airway devices used in anaesthetised rabbits.

This Knowledge Summary has reviewed the available evidence on the use of a SGAD (v-gel®) in rabbit anaesthesia. Since writing a new design of a single use supraglottic airway device (SGAD) has been introduced; currently there is no published evidence on whether this new device has an impact on the risk of injury.

## Summary of the evidence

**Comolli et al. (2020):** 

Population:	Healthy (based on physical examination and health surveillance report) American Society of Anesthesiologists (ASA) score 1, 12 month old female New Zealand White rabbits (*Oryctolagus cuniculus*). The mean body weight ± SD was 2.57 ± 0.15 kg.
Sample size:	14 rabbits.
Intervention details:	General: Rabbits were block randomised into two groups of seven animals each. One group received endoscopic endotracheal intubation intervention (ETI group); the second group received v-gel® supraglottic airway device (VGEL group). Diet: free choice pellets, timothy hay, and water ad libitum. Rabbits were not fasted before anaesthesia. Induction of anaesthesia by intramuscular injection of ketamine at 35 mg/kg and xylazine at 2 mg/kg and meloxicam at 1 mg/kg injected intramuscularly for analgesia. Visual inspection of the airway was performed endoscopically before and after airway device placement. The anaesthesia was maintained via isoflurane and rabbits underwent an ovariohysterectomy then recovered. Airway device placement: Lubricated endotracheal tube (3.5 mm) was placed for the ETI group (n = 7) with the use of an endoscope. Lubricated v-gel® was placed for the VGEL group (n = 7). All the intubations and v-gel® placements were done by the same experienced anaesthetist. Post-mortem: Four days after the anaesthetic event the animals were humanely euthanised by intravenous phenobarbital, tissue samples were collected (proximal larynx, distal larynx, 2 cm distal to larynx, and trachea at the tracheobronchial bifurcation) and fixed in formalin. Four days after fixation the tissue cross-sections were obtained, processed for histopathologic evaluation. The histological evaluation was performed by a blinded board-certified pathologist.
Study design:	Blinded randomised experimental trial study.
Outcome Studied:	Number of attempts and time of airway device placement. Arterial blood gas values (pH, pO2, pCO2, total CO2, HCO3, base excess, and haematocrit (HCT). Histological assessment of proximal larynx, distal larynx, 2 cm distal to the larynx, and trachea at the tracheobronchial bifurcation.
Main Findings (relevant to PICO question):	All animals recovered without signs of irritation to the airway such as coughing. All animals resumed drinking and eating normally within 12 hours after surgery. No animal died from complications and during the post-mortem examination, no animal had signs of aspiration, pneumonia, or pneumonitis. The histological lesions did not show any significant difference in the severity of inflammation, haemorrhage, and necrosis for either larynx or the trachea between the two techniques. However, mild to moderate signs of laryngeal and tracheal trauma was observed in both groups. No statistically significant difference between the two techniques for histopathological lesions to the larynx and tracheal, however, investigators reported that v-gel® caused more trauma to the larynx and epiglottis whereas endotracheal intubation caused more trauma to the trachea.
Limitations:	No explanation of how visual inspection measurements were recorded or analysed in the paper, despite the authors stating that gross visual inspection of the larynx (before and after the airway device placement) underestimated the laryngeal trauma when compared to histological evaluation. The severity of the trauma present in fixed tissue samples is affected by the harvest time post anaesthesia, in this case 4 days. Sampling at multiple time points post anaesthesia would provide a more accurate picture of resulting airway trauma. A small sample size impacts the categorical data analysis and increases the chance of a type II error. The person who performed the placement of the airway devices could not be blinded to the two treatment groups due to the nature of the procedures. Investigators securing airways could be more experienced than a normal general practitioner. Experience could be a factor that might affect the severity of trauma while securing airways.

**Engbers et al. (2017):** 

Population:	Healthy (American Society of Anesthesiologist [ASA] score >2) adult New Zealand White rabbits (n = 12 males, n = 3 females).
Sample size:	15 rabbits.
Intervention details:	General: Block randomised to two treatment groups: Endotracheal tube (ETT) placement group, supraglottic airway device (SGAD) group / v-gel® Number of rabbits allocated to each group is not mentioned. Diet: ad libitum pellets, hay, and water with a small amount of fruit and vegetables. Rabbits were fasted 2 hours prior to the experiment. Animals sedated with dexmedetomidine (0.1 mg/kg) and midazolam (0.5 mg/kg) by intramuscular injection. Anaesthesia was induced with alfaxalone (0.3 mg/kg) intravenously, followed by 1 hour of isoflurane anaesthesia after device placement. Computed tomography (CT) scan of head and neck performed prior to and after the placement of airway devices by a board-certified radiologist. For nine animals (SGAD n = 4, ETT n = 5), arterial blood samples were collected via arterial canula. Airway device placement: Lubricated v-gel® was inserted until further insertion was not possible or the incisors were close to the fixation tabs. If lingual cyanosis was present and was not resolved after three attempts, the rabbit was intubated by endotracheal tube. In the ETT group, the blind technique was used for the insertion of ETT. Each group had a separate investigator for placement of the airway device and investigators conducting device placement received training. Post-mortem: Post anaesthesia all animals were euthanised with an overdose of intravenous sodium phenobarbital. Two hours post euthanasia necropsy was performed and tissue samples (tongue, pharynx, larynx, and trachea) were collected for histological evaluation.
Study design:	Blinded randomised experimental trial study.
Outcome Studied:	Number of attempts and time of placements. Blood pressure (BP), heart rate, respiratory rate, and SpO2. Isoflurane concentration at 15 minutes of anaesthesia. Presence of tongue cyanosis or airway obstructions. Blood gases and electrolyte analysis (SGAD n = 4, ETT n = 5). CT imaging measurements to assess airway diameter and laryngeal compression. Histological assessment of tongue, pharynx, larynx, and trachea.
Main Findings (relevant to PICO question):	Based on histological valuation, significantly greater tracheal mucosal and submucosal trauma was caused by endotracheal intubation compared to v-gel®.
Limitations:	A small sample size impacts the categorical data analysis and increases the chance of a type II error. For histological samples, the tissues were collected 2 hours after euthanasia, this could impact the histological results due to possible post-mortem changes on tissue samples.

**Wenger et al. (2017):** 

Population:	Healthy (on basis of physical examination) female New Zealand White rabbits aged 7 months with a bodyweight of 5.10 ± 0.05 kg (mean ± SD).
Sample size:	10 rabbits.
Intervention details:	Diet: small amount of produce with ad libitum access to water and hay. No fasting prior to the experiment. The produce provided for rabbits is not explained in the paper. All rabbits were anaesthetised four times with 1–2 week intervals in between and each anaesthesia was for insertion of an airway management device (endotracheal tube (ETT), laryngeal mask (LM), supraglottic airway device (v-gel®), and face mask (FM)) in random order. Rabbits sedated with fentanyl citrate (0.05 mg/kg) and fluanisone (1.5 mg/kg) injection intramuscularly and anaesthesia induced with propofol intravenously (1 mg/kg). Anaesthesia was maintained with propofol boluses (1 mg/kg) to allow multiple attempts for airway device placements until placement was achieved. Computed tomography (CT) scans of the head and neck, and abdomen were taken prior to and after controlled manual ventilation (CMV). During recovery meloxicam (1 mg/kg subcutaneously) and Ringer’s lactate fluid (10 ml/kg intravenous [IV] over 30 minutes) were administered.Airway device placement: ETT placed with blind technique and listening to breaths. Lubricated LM (deflated) was inserted until the tube could not be inserted further. Lubricated v-gel® was placed similar to LM. Size R5 was used for rabbits weighing 4.3–5.0 kg and size R6 was used for rabbits weighing more than 5 kg. FM was placed tightly around the nose and mouth and taped around the head. Placement of all devices was performed by board-certified anaesthetists or an experienced veterinarian. Controlled manual ventilation (CMV): Rocuronium (0.3 mg/kg IV) was administered to induce apnoea and CMV (respiratory rate of 30 breaths/min) was performed for 4 minutes, followed by administration of sugammadex (4 mg/kg) before stopping CMV and allowing spontaneous ventilation.
Study design:	Randomised crossover experimental trial.
Outcome Studied:	Dose of propofol, time, and number of attempts for device placement. Presence of swallowing during placement or apnoea. Amount of leakage from each airway device during spontaneous ventilation (SV) and CMV. Cardiorespiratory values every 5 minutes. The Peak Inspiratory Pressure if the leak was >25%. CT scan measurements for: LM and v-gel® positions; Presence of compression to larynx with LM, v-gel® placement during SV; Presence of gastric tympanism induced by CMV.
Main Findings (relevant to PICO question):	With the placement of v-gel®, lingual cyanosis was reported in several rabbits, with resolution 30 seconds after removal of the device. The study fails to report the exact number of rabbits that had lingual cyanosis after placement of v-gel®. V-gel® placement caused severe laryngeal compression in one rabbit. Based on CT imaging in 5/10 cases v-gel® was positioned correctly. In two of these moderate laryngeal compressions with mucous accumulation was detected and in one case severe laryngeal compression was detected. In 2/10 cases v-gel®, resulted in hypoxaemia due to severe laryngeal compression improper placement. The height and width of the larynx were significantly smaller with the placement of v-gel® compared to LM and FM.
Limitations:	A small sample size impacts the categorical data analysis and increases the chance of a type II error. Multiple investigators were assigned for the placement of the airway. It is not clear if a specific group was allocated to a specific investigator. The experience of investigators could impact the results. This study mainly focuses on rabbits above body weight of 5 kg and discuss v-gel® size for body weight above 5 kg. However, in a clinical setting different breed of rabbits with smaller body weight can be presented and further research is needed to work on v-gel® sizes appropriate for smaller breed of rabbits.

## Appraisal, application and reflection

The increasing popularity of pet rabbits and the willingness of owners to pay for veterinary services (Mayer et al., 2017) means that more rabbits are presenting to veterinary practices for care. Veterinary practices need to accommodate the medical and welfare needs of rabbit patients and provide the necessary services, including general anaesthesia for surgical procedures such as ovariohysterectomy. General anaesthesia in rabbits has higher risks and higher mortality rates compared to cats and dogs (Brodbelt et al., 2008). The main factors contributing to this high risk are difficulty establishing a secure airway, underlying respiratory diseases, and cardiorespiratory complications during anaesthesia (Brodbelt et al., 2008; and Eatwell, 2014).

Securing the airway in rabbits is made difficult due to their unique airway anatomy. Rabbits have small and narrow mouths with large incisors, large molars and large tongues relative to their skull size, which makes it difficult to visualise the larynx for intubation (Donnelly & Vella, 2020). The most common methods used to secure the airway in rabbits are endotracheal intubation and placement of a well-fitting face mask. However, both endotracheal tubes and face masks have disadvantages that can impact the risk of anaesthesia (Eatwell, 2014). Face masks allow delivery of oxygen and anaesthetic gases but do not guarantee airway patency, thus can cause clinically significant hypoxaemia and hypercapnia. Endotracheal intubation can cause mucosal damage to the upper airways, leading to swelling and potential narrowing (Bateman et al., 2005; and Hawkins & Pascoe, 2021). Endotracheal intubation also requires significant training and practice. To address these issues, supraglottic airway devices (SGAD), similar to those used in human medicine, have been introduced and trialled as an alternative airway management device for rabbits. The use of a human supraglottic airway device was tested in rabbits and reported to cause lingual cyanosis, possibly due to pressure on the lingual vasculature (Crotaz, 2013; and Kazakos et al., 2007). In recent years, a new rabbit-specific SGAD (v-gel®) has been designed to decrease the risk of complications observed with the use of a human SGAD.

The three studies included in this critical appraisal reported varied outcomes. Significant limitations were identified for all three studies, including small sample size, use of multiple investigators for device placement and the use of rabbit breeds that differ in size from breeds commonly kept as pets.

Comolli et al. (2020) and Engbers et al. (2017) both compare the placement of v-gel® and an endotracheal tube (ETT) in rabbits. However, the findings of the two studies are incongruent. Comolli et al. (2020) concluded that overall, the trauma caused by v-gel® and ETT placement is not significantly different. The authors report that v-gel® caused more injury to the larynx and epiglottis, and ETT caused more injury to the trachea, although the differences between the devices were not statistically significant. In contrast, Engbers et al. (2017) identified that ETT causes more significant injury to tracheal mucosal and submucosal airways compared to v-gel®. The different findings in these two studies could be due to differences in ETT placement method, given that Comolli et al. (2020) used endoscopic guided endotracheal intubation, while Engbers et al. (2017) performed a blind technique for placement of ETT. Another possible explanation could be the experience of investigators placing the devices. Goldmann & Ferson (2005) report that training for airway management by anaesthetists can reduce anaesthesia-related morbidity and mortality. The third study (Wenger et al., 2017) compared two additional airway devices, laryngeal mask, and face mask, in addition to v-gel® and ETT. Wenger et al. (2017) mainly studied the amount of laryngeal compression caused by the different devices and found that v-gel® compressed the larynx more significantly than laryngeal mask and face mask. This study did not look at the direct injury to the upper airways, but it was assumed that compression of the larynx would lead to airway damage, and therefore was relevant to the PICO question.

The methods used to evaluate airway injury were similar between the Comolli et al. (2020) and Engbers et al. (2017) studies. Both papers performed histological evaluation of post-mortem samples following anaesthesia. However, the time of sample collection differed between the studies. In Comolli et al. (2020) study samples were collected 4 days after anaesthesia. In Engbers et al. (2017) study samples were collected 2 hours after anaesthesia. The different time intervals between device placement and sample collection could impact the observed histological changes to the tissues. Collection after 2 hours would show any acute injury changes, such as acute inflammation and damage to tissue integrity, but not long-term damage such as chronic inflammatory infiltrate, fibrosis or stricture formation which could take days or weeks to develop. On the other hand, collection of tissue after 4 days may fail to identify the extent of the acute inflammatory changes immediately following the injury. If the tissue damage is significant but not severe enough to cause chronic change, it may still cause airway complications during the recovery period with resolution prior to sample collection at day 4 (Anderson, 2013).

The level of experience of the investigators performing the placement of each airway device differed between the three studies. In the two blinded studies (Comolli et al., 2020; and Engbers et al., 2017) the pathologist evaluating the tissue histology was blinded to treatment, however, the investigators placing the airway devices could not be blinded due to the nature of the procedure. This could impact the technique used by the investigator due to unconscious bias about which device was superior. Moreover, in the Engbers et al. (2017) study the investigator was more experienced using ETT compared to the v-gel®. In Wenger et al. (2017) the level of experience with device placement was not clear as multiple investigators were involved. One limitation that stood out in the Comolli et al. (2020) study is the use of visual inspection of upper airway. The authors do not describe how the measurements were recorded and analysed for visual inspections of the upper airways. Finally, it should be considered that the studies included only one breed of rabbit, that does not reflect the common pet rabbit breeds.

Considering the findings and limitations of each study, there is currently insufficient evidence to confirm that the use of v-gel® during general anaesthesia in rabbits causes less injury to the upper airways compared to other airway management devices. More research such as randomised blinded clinical trial with larger sample size can be helpful to learn if v-gel® use in rabbits is beneficial to them routinely in the clinical setting, given that less damage to the upper airways, will lead to better recovery from anaesthesia and fewer complications.

## Methodology Section

**Search Strategy:** 

Databases searched and dates covered:	CAB Abstracts via Web of Science (1910–present)Scopus (1960–present)Web of Science Core Collections (1900–present)Medline (PubMed) (1900–present)
Search strategy:	CAB Abstracts and Web of Science Core Collections: (Rabbit OR Rabbits OR *Oryctolagus cuniculus)* (Anaesthesia OR general anaesthesia) (V-gel OR Supraglottic airway device OR SGAD) (Endotracheal tube OR tracheal tube OR endotracheal intubation OR airway OR mask OR complications OR laryngeal tube) (((1 AND) 2 AND) 3 AND) 4 Scopus: (Rabbit OR Rabbits OR *Oryctolagus-cuniculus)* (Anaesthesia OR general-anaesthesia) (V-gel OR Supraglottic-airway-device OR SGA) (Endotracheal-tube OR tracheal-tube OR endotracheal-intubation OR airway OR mask OR complications OR laryngeal-tube) #1 AND #2 AND #3 AND #4 PubMed:(((Rabbit OR Rabbits OR Oryctolagus cuniculus) AND (Anaesthesia OR general anaesthesia)) AND (V-gel OR Supraglottic airway device OR SGA)) AND (Endotracheal tube OR tracheal tube OR endotracheal intubation OR airway OR mask OR complications OR laryngeal tube)
Dates searches performed:	27 May 2022

**Exclusion / Inclusion Criteria:** 

Exclusion:	Papers written in English. Case series, case control studies, clinical trials and systemic reviews. Study population: rabbits undergoing general anaesthesia. Intervention: use of v-gel®. Comparator: other airway control devices. Outcomes: include measures of airway trauma or damage or inflammation.
Inclusion:	Case studies, narrative reviews, opinion pieces, methods papers, non-English publications.

**Search Outcome:** 

Database	Number of results	Excluded – It did not include rabbits	Excluded – Non-English papers	Excluded – Did not focus on v-gels®	Excluded – Did not focus on trauma / injury / damage to the airways	Excluded – Case studies, narrative reviews, opinion pieces, methods papers	Excluded – Duplicates	Total relevant papers
CAB Abstracts	18	1	1	5	4	4	0	3
Web of Science	10	2	0	1	2	0	5	0
PubMed	16	1	0	4	2	1	8	0
Scopus	18	0	0	0	0	0	18	0
Total relevant papers when duplicates removed								3

## Acknowledgements

I would like to dedicate this paper in memory of my professor Dr Frazer Allan. I would also like to thank Sanaa Zaki for holding my hand throughout this project.

## ORCID

Jasmine Gheini: https://orcid.org/0000-0002-7243-6239

Sanaa Zaki: https://orcid.org/0000-0002-6393-6055

## Conflict of Interest

The authors declare no conflicts of interest.
