# Erratum to: Does the use of supraglottic device in rabbits cause less injury than other airway management devices?

**DOI:** 10.18849/ve.v8i2.663

**Published:** 2023-05-05

**Authors:** Jasmine Gheini+, Sanaa Zaki+

**Keywords:** ANAESTHESIA, RABBIT, INTUBATION, ENDOTRACHEAL TUBE, TRAUMA, INJURY, AIRWAY MANAGEMENT, V-GEL®, SUPRAGLOTTIC AIRWAY DEVICE, FACE MASK, LARYNGEAL MASK

## Abstract

The original article was published in Veterinary Evidence Vol 7, Issue 4 (2022): https://doi.org/10.18849/ve.v7i4.608

Unfortunately the original version of the article was missing the following statement.

This Knowledge Summary has reviewed the available evidence on the use of a SGAD (v-gel®) in rabbit anaesthesia. Since writing a new design of a single use supraglottic airway device (SGAD) has been introduced; currently there is no published evidence on whether this new device has an impact on the risk of injury.

This error was in both the HTML and PDF versions. This has now been updated in both the HTML and PDF versions, and can be found in the clinical bottom line and the evidence section.

## Erratum

Unfortunately the original version of the article was missing the following statement.

This Knowledge Summary has reviewed the available evidence on the use of a SGAD (v-gel®) in rabbit anaesthesia. Since writing a new design of a single use supraglottic airway device (SGAD) has been introduced; currently there is no published evidence on whether this new device has an impact on the risk of injury.

This error was in both the HTML and PDF versions. This has now been updated in both the HTML and PDF versions, and can be found in the clinical bottom line and the evidence section.

## Disclaimer

Knowledge Summaries are a peer-reviewed article type which aims to answer a clinical question based on the best available current evidence. It does not override the responsibility of the practitioner. Informed decisions should be made by considering such factors as individual clinical expertise and judgement along with patient’s circumstances and owners’ values. Knowledge Summaries are a resource to help inform and any opinions expressed within the Knowledge Summaries are the author's own and do not necessarily reflect the view of the RCVS Knowledge. Authors are responsible for the accuracy of the content. While the Editor and Publisher believe that all content herein are in accord with current recommendations and practice at the time of publication, they accept no legal responsibility for any errors or omissions, and make no warranty, express or implied, with respect to material contained within. For further information please refer to our Terms of Use.

